# Involvement of acid sensing ion channel (ASIC)-3 in an acute urinary bladder-colon cross sensitization model in rodent

**DOI:** 10.3389/fpain.2023.1083514

**Published:** 2023-03-08

**Authors:** Karim Atmani, Mathieu Meleine, Ludovic Langlois, Moïse Coëffier, Pablo Brumovsky, Anne-Marie Leroi, Guillaume Gourcerol

**Affiliations:** ^1^ Nutrition, Gut & Brain Unit (INSERM U1073), Institute for Biomedical Research and Innovation, Rouen University, Rouen, France; ^2^ Institute of Research in in Translational Medicine, CONICET-Austral University, Pilar, Argentina; ^3^ Department of Physiology, Rouen University Hospital, Rouen, France

**Keywords:** cross-organ sensitization, acid sensing ion channel-3, urinary bladder, colonic sensitivity, hypersensitivity

## Abstract

**Introduction:**

Irritable bowel syndrome and bladder pain syndrome are both characterized by pain in response to organ distension. Epidemiologic studies showed that these two syndromes are often overlapped. Such overlap may be due to sharing of common extrinsic innervations between the colorectum and the urinary bladder, where cross-sensitization of the urinary bladder and the colon would occur in response to mechanical distension of either organ. The aim of this project was to develop and characterize a rodent model of urinary bladder-colon sensitization and to assess the role of the acid sensing ion channel (ASIC)-3.

**Methods:**

Double retrograde labelling was performed to identify extrinsic primary afferent neurons innervating both the colon (Fluororuby) and urinary bladder (Fluorogold) in the L6-S1 dorsal root ganglia (DRG) in Sprague Dawley rats. The phenotype of the colon/urinary bladder co-innervating primary afferent neurons was assessed using immunohistochemistry directed against ASIC-3. Cross-organ sensitization was induced in Sprague Dawley rats by using an echography-guided intravesical administration of acetic acid (0.75%) under brief isoflurane anesthesia. Colonic sensitivity was assessed in conscious rats by measuring abdominal contraction during isobaric colorectal distension (CRD). Measurement of urinary bladder and colonic paracellular permeabilities and tissue myeloperoxidase assay were performed. The involvement of ASIC-3 was assessed by use of S1 intrathecal administration of the ASIC-3 blocker, APETx2 (2.2 µM).

**Results:**

Immunohistochemistry showed that 73.1% of extrinsic primary afferent neurons co-innervating the colon and the urinary bladder express ASIC-3. By contrast, extrinsic primary afferent neurons innervating the colon only or the urinary bladder only were positive for ASIC-3 in 39.3% and 42.6%, respectively. Echography-guided intravesical administration of acetic acid resulted in colonic hypersensitivity to colorectal distension. This effect started 1 h post-injection and lasted up to 24 h, and was not longer seen after 3 days after injection. No colonic hyperpermeability and no difference in urinary bladder and colon MPO activity was observed between control and acetic acid-treated rats. Colonic sensitization by intravesical acetic acid administration was prevented by S1 intrathecal administration of APETx2.

**Conclusion:**

We developed an acute pelvic cross-organ sensitization model in conscious rat. In this model, cross-organ sensitization is likely to involve S1-L6 extrinsic primary afferents co-innervating the colon and urinary bladder through an ASIC-3 pathway.

## 1. Introduction

Experimental and clinical studies on irritable bowel syndrome (IBS) and bladder pain syndrome (BPS) highlight the close relationship between digestive symptoms and urinary symptoms. Indeed, clinical studies point out that a high prevalence of urinary symptoms are found in patients with IBS, including urinary bladder syndrome or overactive urinary bladder. Similarly, among patients with BPS, 52% share IBS criteria, while correspondingly, 40%–60% of IBS patients also fulfill diagnostic criteria for BPS (1–3). The main hypothesis to explain this association relies on alteration of visceral and/or somatic perception. This is supported by the fact that urinary bladder and colon may share common afferent nerves, and the information is conveyed through the same central anatomical structures. Recently, the phenomenon of cross sensitization has been acknowledged and is based on the convergence of neuronal afferent fibers, both at the level of dorsal root ganglia (DRG) and spinal/supraspinal structures (4). Studies in rats have confirmed that acute urinary bladder sensitization decreases the thresholds of colonic sensitivity associated with colorectal distension (CRD) and, conversely, acute colonic sensitization induces an increase in the frequency of urinary bladder contractions (5). It was also shown after acute colonic sensitization an increase in sodium channel activity in the C fibers of the pelvic nerve innervating the urinary bladder. However, these same C fibers are blocked by capsaicin injected in the urinary bladder, resulting in inhibition of cross sensitization (6). These results testify to the direct role of afferent neurons in this mechanism. However, these models were using inflammatory insults, while IBS and BPS are known to be unrelated to visceral inflammatory processes.

The target organs of primary afferent endings produced by DRG neurons has been identified in animals by retrograde labelling studies (7, 8). It was shown that the neuronal bodies of primary afferents targeting the colorectum or the urinary bladder are predominantly present in lumbar and sacral DRGs (9). In addition, it has been shown that some DRG neurons target both organs, thus transmitting signals arising from both the colorectum and the urinary bladder (10). For instance, 17% of the neurons in the lumbosacral DRG innervate both the colon and urinary bladder in the mouse (11). It has been shown that acid receptors such as acid sensing ion channels (ASICs) and transient receptor potential channels (TRPs) are expressed by primary afferent neurons innervating the colon and urinary bladder (12–14). Finally, in acute models of urinary bladder or bowel hypersensitivity, neuronal hyperexcitability has been observed in DRG neurons in response to a nociceptive stimulus (15, 16).

To better characterize mechanisms involved in acute cross-organ sensitization, we aimed to develop a non-inflammatory rat model of colorectal hyperalgesia induced by acidic stimulation of the urinary bladder. Considering the fact that subpopulations of primary afferent neurons have been shown to express acid-sensing ion channels, we investigated whether those co-innervating the colorectum and the urinary bladder do express ASIC-3 channels, and whether ASIC-3 channels may be involved in acid induced acute cross-organ sensitization.

## 2. Materials and methods

### 2.1. Animals

Male Sprague-Dawley rats (250–350 g, 40–52 days old; Janvier, Le Genest-St-Isle, France) were housed in a temperature-controlled environment (22°C) with a 12-h light/dark cycle. The rats had free access to standard rat chow (RM1 diet; SDS, Witham, Essex, UK) and drinking water. The protocol was approved by the local Committee on the Ethics of Animal Experiments (Ethical agreement Number: N/02-01-13/02/01-16) and these experiments were adhered to the IASP and SFN guidelines for research using animal subjects.

### 2.2. Double retro-labelling

Surgery was performed in anaesthetized rats using sodium ketamine (100 mg/kg) and xylazine (Rompun 2%; 10 mg/kg), given intraperitoneally (i.p.). After laparotomy, Fluorogold (FG, *Hydroxystilbamidine, methanesulfonate*, 4%, 1 µl, 10 injections; Interchim, Montluçon, France) and Fluororuby (FR, *Dextran, Tetramethylrhodamine, 10000 MW, Lysine Fixable*, 10%, 1 µl, 10 injections; Interchim, Montluçon, France) (17) were injected in the all part of the urinary bladder wall and in the wall of the colorectum segment respectively. Seven days after these injections, the L6 and S1 DRGs were extracted after intracardiac perfusion using Tyrode solution (NaCl 6.8 g; KCl 0.4 g; MgCl_2_ × 6 H_2_O 0.15 g; MgSo4 × 7H_2_O 0.1 g; NaH_2_PO_4_ 2H_2_O 0.19 g; Glucose 1 g; NaHCO_3_ 2.2 g; Fill up to 1l with H_2_O), followed by a fixation solution composed of 4% paraformaldehyde (Sigma) and 0.3% picric acid (Sigma) in 300 ml of 0.1 M phosphate buffer. After overnight incubation in fixation solution, DRGs were further incubated in sucrose (10%, 20% and 30%) at 4°C during 24 h each one. This was followed by inclusion in TissueTek®, freezing in isopentan at −40°C, and cryostat sectioning after 24 h at −80°C (LEICACM1950, Ruel-Malmaison, France; 20 µm). A fluorescent microscope was used for identification of colorectum-only, urinary bladder-only and colorectum-urinary bladder co-innervating DRG neurons.

### 2.3. ASIC3 immunofluorescence

The expression of ASIC3 in retrogradely labelled DRG neurons was assessed by immunofluorescence. Tissue sections were first incubated with an anti-ASIC3 antibody from the rabbit (from Alomone, diluted at 1:300) overnight at 4°C. This was followed by 1 h incubation with a goat anti-rabbit secondary antibody conjugated with Alexa-488 (from Invitrogen, diluted at 1:300), three washes in PBS and coversliped using glycerol/DABCO mounting media. Pictures were taken with a Leica photomicroscope at x10 magnification.

### 2.4. Acetic acid cross-sensitization

In anesthetized rats (Isoflurane: 3% in 1.5 L/min of air), an intra-urinary bladder acetic acid instillation (0.75%, 500–700 µl) was performed under ultrasound monitoring. Control rats were injected with saline solution (NaCl 0.9%). The use of the ultrasound system allowed us to confirm the injections in the urinary bladder of the rats by a visual control (Supplementary Material). The rat woke up immediately after brief anesthesia and was left undisturbed for at least 1 h before any experiment.

### 2.5. Colorectal distension

A spherical infinitely compliant distension balloon (diameter: 2 cm) was made using a polyethylene bag attached to a 5 mm diameter polyethylene catheter (Dutscher, Brumath, France) drilled in its extremity. The balloon was inserted in the colorectum of anaesthetized rats, secured to the tail with tape and connected to an electronic barostat (G&J Electronics Inc, Toronto, Canada) to perform isobaric graded colorectal distensions (CRD).

### 2.6. Visceral sensitivity measurement

**Figure 1:**
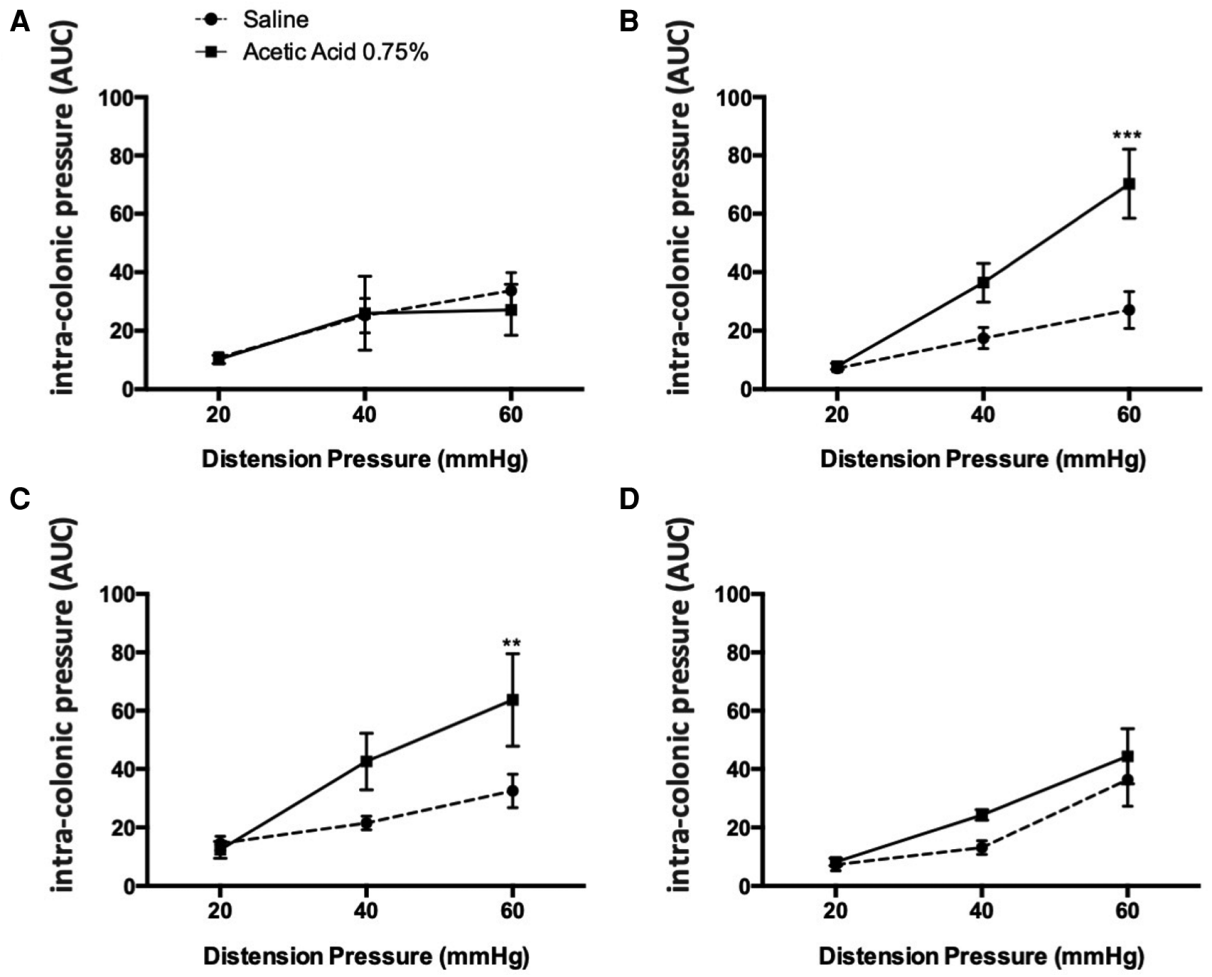
Kinetics of the effect of an acetic acid injection on colonic sensitivity. Colonic sensitivity assessment by CRD, 30 min (**A**), 1 h (**B**), 3 days (**C**) and 7 days (**D**) after acetic acid injection at 0.75% (*n *= 6–9 per group; *p < 0.05, ***p < 0.001, Two-way ANOVA followed by Bonferroni *post-hoc* test).

Colorectal sensitivity was measured in awake rats 60 min, 1 day, 3 days and 7 days following acetic acid administration. Visceral pain was assessed by continuous monitoring of pressure changes resulting from abdominal wall contractions induced by CRD. A miniaturized pressure transducer catheter (SPR-524 Mikro-Tip catheter; Millar Instruments, Houston, TX) with a polyethylene balloon lubricated with medical grade lubricant was introduced into the colon such that the middle of the pressure sensor (3.5 F) was 2 cm proximal to the anus. The catheter was then secured to the tail with tape, and colonic contractions were recorded in conscious rats immediately after their placement in the restraint tube. Changes in intracolonic pressure (ICP), reflecting viscero-motor responses, were used as a surrogate marker of colorectal sensitivity (18). Variation of intracolonic pressures was quantified during graded CRD at 20, 40 and 60 mmHg (Figure 1). Each distension pressure was applied twice for 20s at 4-min intervals.

### 2.7. Measurement of urinary bladder and colonic paracellular permeabilities

Urinary bladder and distal colon samples were removed and cut along the mesenteric border. Urinary bladder and colonic permeabilities were assessed by measuring FITC-dextran (4 kDa) fluxes in Ussing chambers with an exchange surface of 0.07 cm^2^ (Harvard Apparatus, Holliston, MA). FITC-dextran (5 mg/ml) was placed in the mucosal side. After 3 h at 37°C, medium from the serosal side was removed and stored at −80°C. The fluorescence level of FITC-dextran (excitation at 485 nm, emission at 535 nm) was measured in 96-well black plate with spectrometer Chameleon V (Hidex Co, Turku, Finland). Values were converted to concentration (mg/ml) using a standard curve (19).

### 2.8. Tissue myeloperoxidase assay

Myeloperoxidase (MPO) was measured in colonic and in urinary bladder tissues. A piece of colon or of urinary bladder (around 50 mg) was thoroughly washed in PBS and homogenized (50 mg/ml) in 0.5% hexadecyltrimethylammonium bromide (Sigma) in 50 mmol/L PBS, (pH 6.0), freeze-thawed 3 times, sonicated and centrifuged. The MPO was assayed in the supernatant by adding 1 mg/ml of dianisidine dihydrochloride (Sigma) and 5 × 10%–5 × 4% H_2_O_2_ and the change in optical density measured at 450 nm. Human neutrophil MPO (Sigma) was used as standard. One unit of MPO activity was defined as the amount that degraded 1.0 µmol of peroxide/min at 25°C (20).

### 2.9. FR, FG, FR and FR DRG neuron and ASIC-3-expressing DRG neuron quantifications

The quantifications of FR neurons, FG neurons, FR and FG neurons and ASIC-3 positive neurons in L6 and S1 DRGs were done on 4–5 sections per DRG using Fiji-ImageJ software (*n* = 5–8 rats). The percentage of convergent neurons was calculated from the total sum of single FR and FG labelled cells (taken together as 100%).

### 2.10. Intrathecal injection of an ASIC3 blocker

APETx2, an ASIC3 blocker (2.2 µM, 25 µl) (21) or saline solution were administered by acute intrathecal injection (using a 25G needle and an 50 µl Hamilton syringe) between L6 and S1, 1 min before acetic acid injection at 0.75% in the urinary bladder (*n* = 6 per group). Colorectal sensitivity was assessed, 60 min after acetic acid injection, as described before.

### 2.11. Statistical analysis

All data were expressed as mean ± SE. Statistical analyses were performed with GraphPad Prism software. For the CRD analysis in model validation, a two-way (Volume and Treatment) ANOVA followed by Bonferroni *post-hoc* test for multiple comparisons were used. For the assessment of ASIC-3 positive cells in L6 and S1 DRGs, a Kruskal-Wallis test followed by a Dunn *post-hoc* test for multiple comparisons were used. A *p* value less than 0.05 was considered statistically significant.

## 3. Results

### 3.1. Development of a cross-organ sensitization model using intravesical injection of acetic acid

Intravesical injection of acetic acid at 0.75% under ultrasound monitoring did not induce colonic hypersensitivity within 30 min following the administration since no difference in intraluminal pressure variation in response to colorectal distensions of 20, 40 and 60 mmHg was observed between control and treated animals (Figure 1A). In contrast, 1 h after injection, an increase of the colonic nociceptive response during colorectal distension occurred at 60 mmHg (*p* < 0.001; Figure 1B). This hypersensitivity lasted up to 3 days after intravesical injection of acetic acid (Figure 1C). By contrast, 7 days after the injection, the colonic nociceptive response during CRD in treated animals by intravesical acetic acid returned to values comparable to those of control animals (Figure 1D).

### 3.2. Intestinal permeability

**Figure 2:**
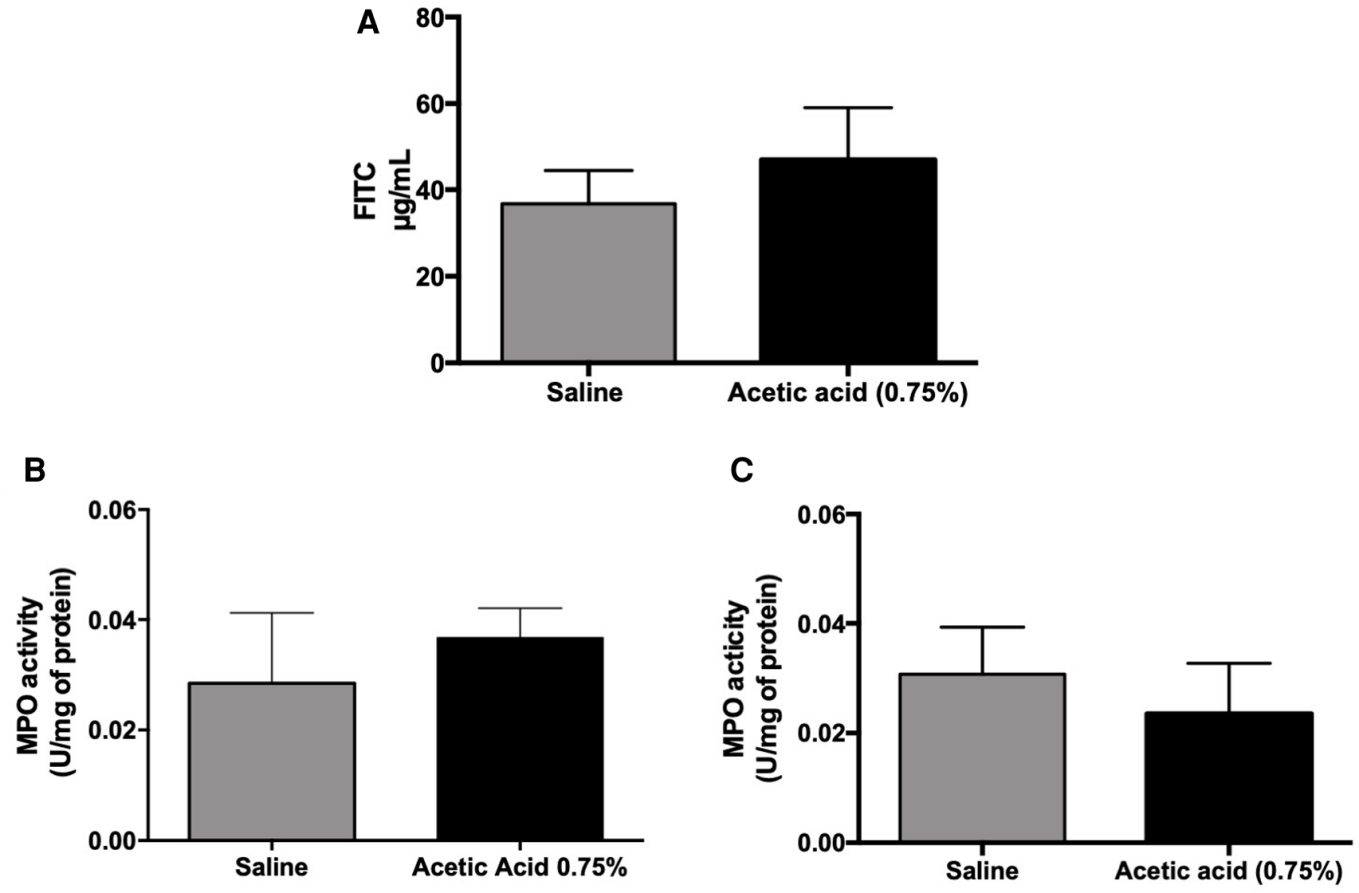
Intestinal permeability and MPO activity measurements in cross-hypersensitization model induced by acetic acid (0.75%). One hour after acetic acid intra-urinary bladder injection (0.75%) under ultrasound monitoring, the intestinal permeability was assessed by the Ussing chamber technique using FITC-Dextran as a marker of the permeability (**A**). Urinary bladder (**B**) and colonic (**C**) myeloperoxidase activity were measured in the tissues 1 h after acetic acid injection in the urinary bladder (*n* = 5 per group; Mann–Whitney test).

Fluorescence quantification on the serosal side of the Ussing chamber showed no significant difference in the passage of FITC-Dextran from the mucous to the serosal side of the colorectal wall of the group receiving intravesical acetic acid compared to the control group (Figure 2A).

### 3.3. Myeloperoxidase activity

No difference in MPO activity was observed between control and acetic acid-treated rats, both in urinary bladder and colon samples (Figures 2B,C).

### 3.4. Identification and ASIC-3 characterization of extrinsic primary afferent neurons co-innervating the urinary bladder and colorectum by double retrograde labelling

**Figure 3:**
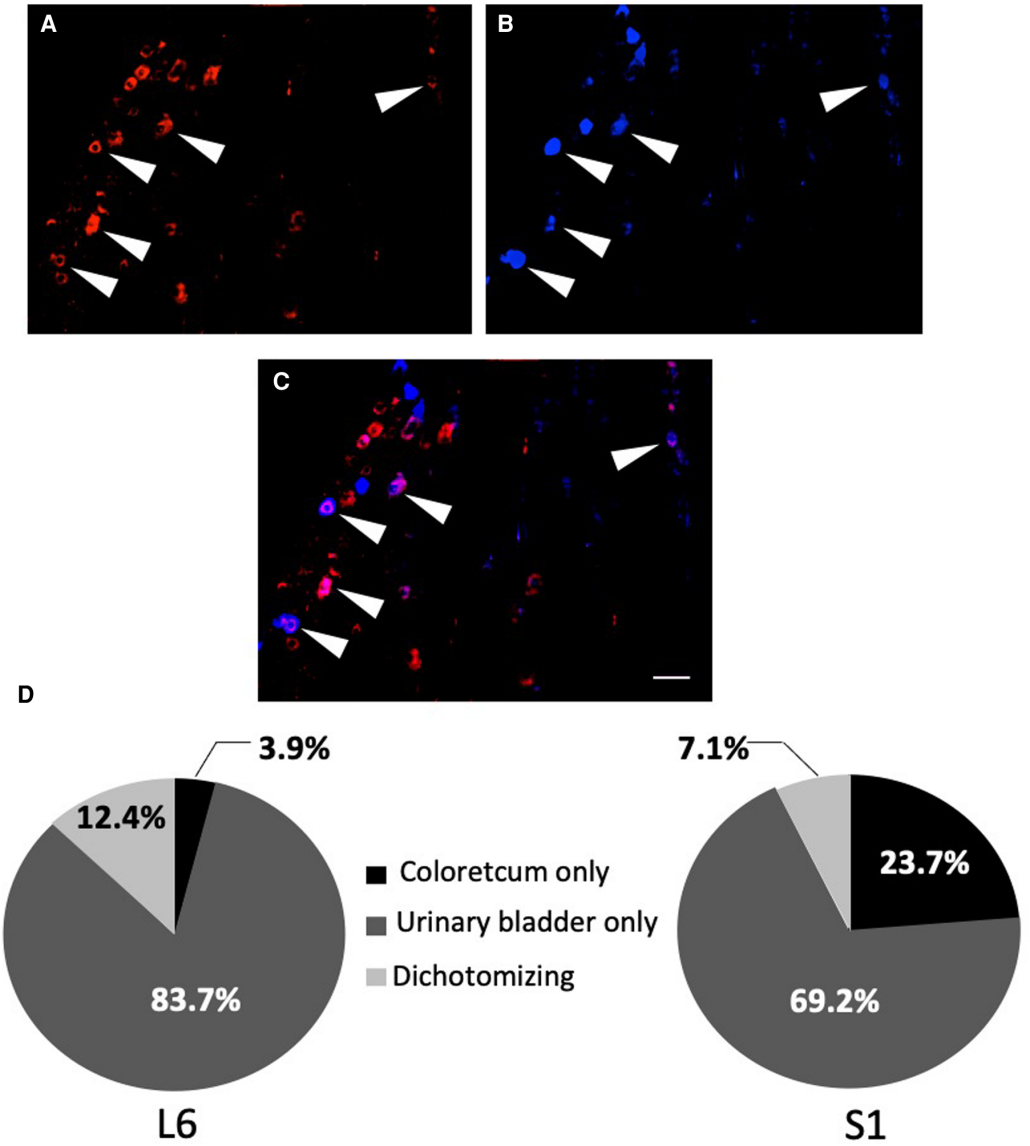
Percentage of urinary bladder neurons-only, colorectal neurons-only and dually-projecting urinary bladder and colonic neurons in L6 and S1 DRGs. Percentage was determined by double retrograde labelling, using fluororuby (FR; **A**) in red for the colon, fluorogold (FG; **B**) in blue for the urinary bladder and fluororuby with fluorogold (FR + FG; **C**) in purple for the colon and the urinary bladder. The arrows show the primary afferent neurons co-innervating the colon and the urinary bladder (scale bar = 100 µm). Quantification was done on six L6 and S1 DRG sections (*n* = 6–8; **D**).

Injection of FG into the wall of the urinary bladder and FR into the wall of the colorectum in the rat showed that there was an average 9.7% of retrogradely traced neurons (FG only, FG only and FG + FR neurons; Figures 3A–C) within the L6 and S1 DRGs that co-innervating the urinary bladder and colon (Figure 3D). 13.8% innervating the colorectum only and 76.5% innervating the urinary bladder only (Figure 3D).

**Figure 4:**
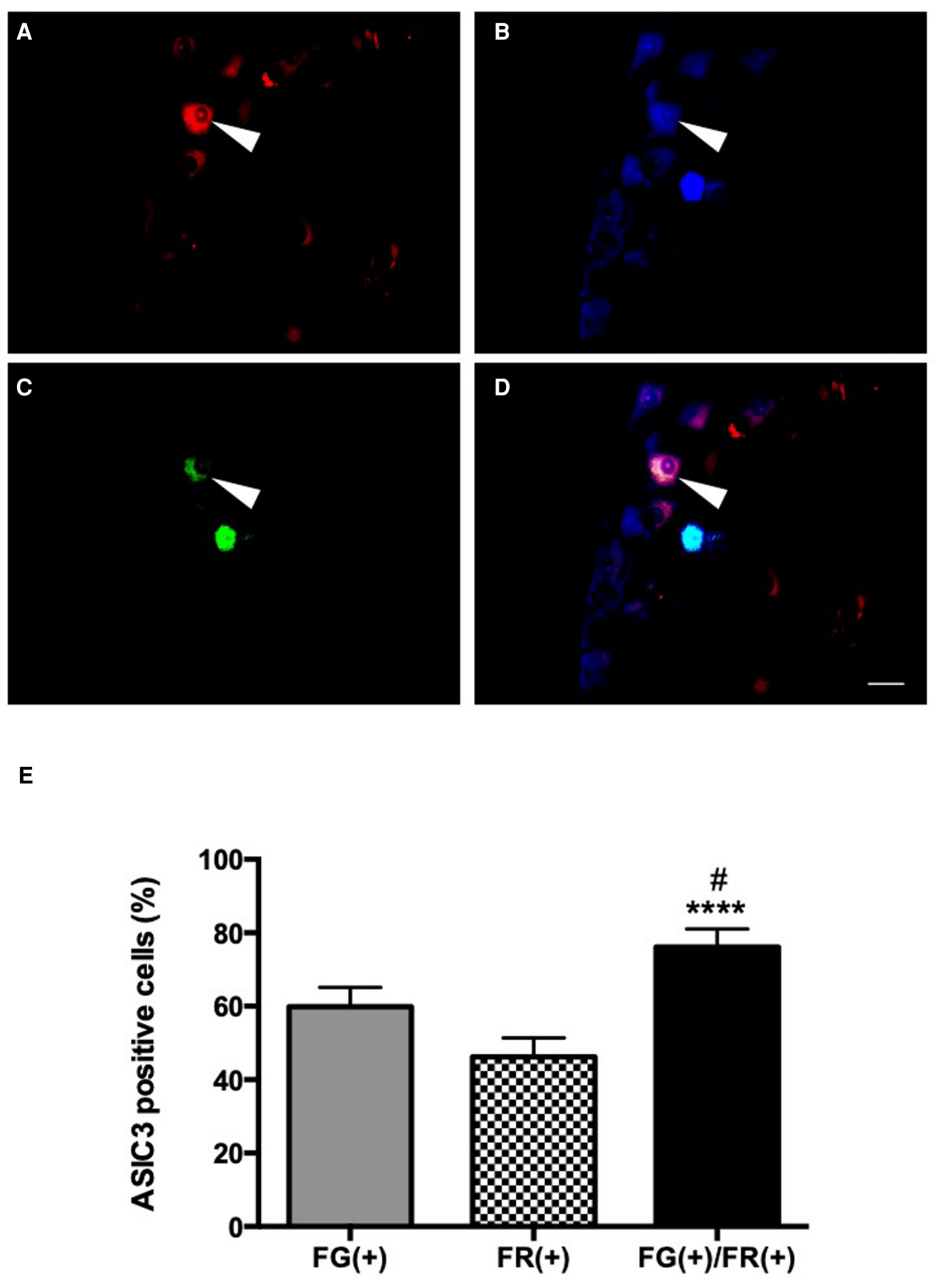
ASIC3 expression in dually-projecting urinary bladder/colorectal L6 and S1 DRG neurons. Colorectal primary afferent neurons expressing FR (red, **A,D**) and urinary bladder primary afferent neurons expressing FG (blue, **B,D**). ASIC3 positive neurons in green (FITC, **C,D**). Triple labelling is observed in **D**, by overlapping of the three fluorochromes (scale bar = 50 µm). The arrow shows a primary afferent neuron co-innervating the colon and the urinary bladder expressing ASIC3. Average of the percentage cells innervating the urinary bladder, the colon and co-innervating the urinary bladder and the colon, expressing ASIC3 in L6 and S1 DRGs (**E**). Percentage of urinary bladder primary afferent-only neurons expressing ASIC3 [FG(+)], colorectal primary afferent-only neurons expressing ASIC3 [FR(+)], or dually-projecting urinary bladder/colonic primary afferent neurons expressing ASIC3 [FG(+)/FR(+)]; (*n* = 5; *p < 0.05 et ****p < 0.0001 [compare to FR(+)]; #*p* < 0.05 [compare to FG(+)]; Kruskal-Wallis test followed by Dunn *post-hoc* test).

Retrograde labeling also allowed for the identification of 46.2%, 59.8% and 76.1% colorectal (Figure 4A), urinary bladder (Figure 4B) and dually-projecting DRG neurons (Figure 4D), respectively, expressing ASIC-3 (*p* < 0.0001 vs. colon only and *p* < 0.05 vs. urinary bladder only; Figures 4C–E).

### 3.5. Effect of the ASIC-3 blocker on cross sensitization

**Figure 5:**
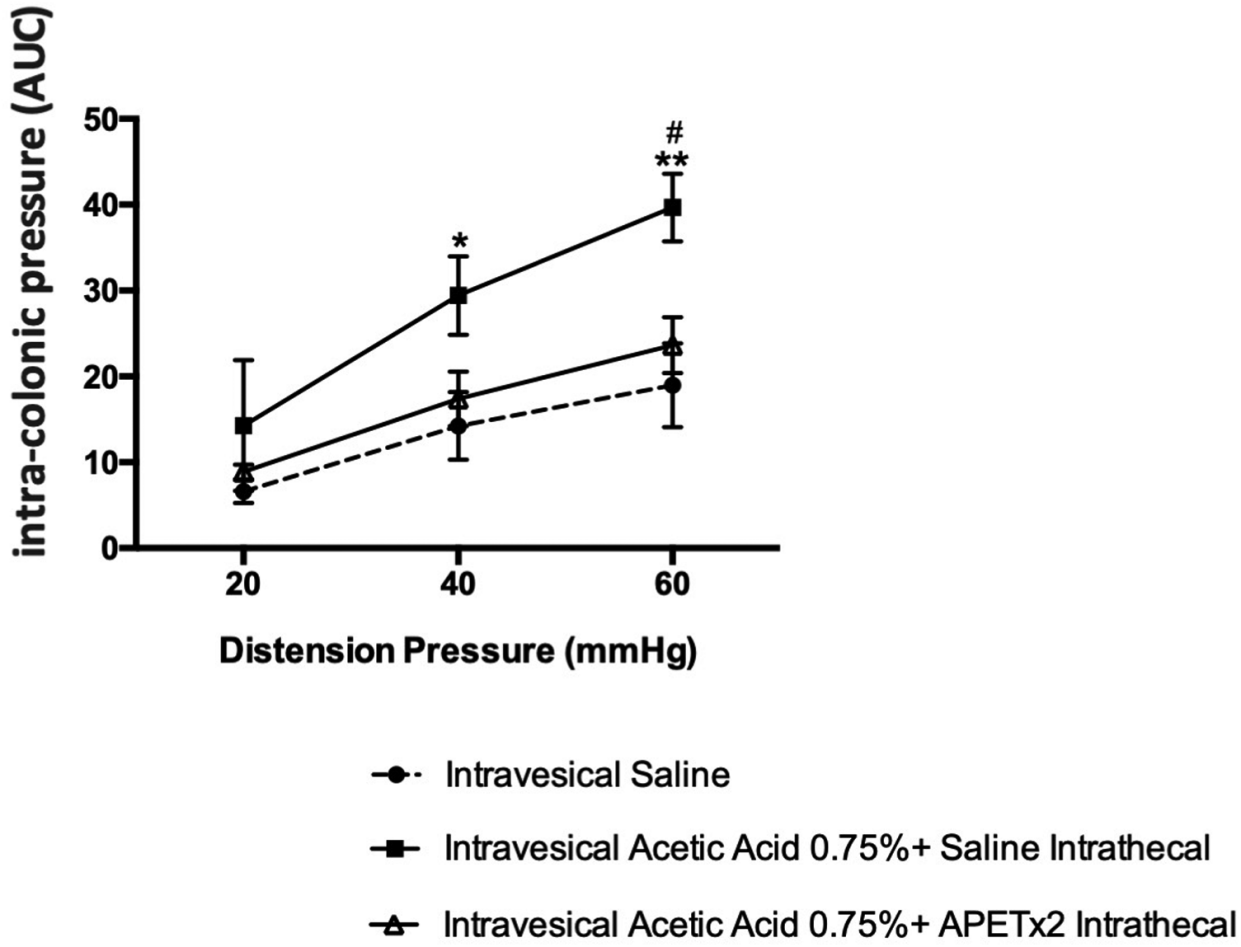
Effect of an ASIC3 blocker by intrathecal injection on cross-organ sensitization. Spinal inhibition of ASIC3 by intrathecal injection of APETx2 decreased the colonic hypersensitivity, assessed 60 min after acetic acid injection in the bladder (*n* = 6/group; *p < 0.05, **p < 0.01).

Blocking of ASIC-3 channels by intrathecal injection of APETx2 (2.2 μM) reduced the viscero-motor responses to CRD (Figure 5). Indeed, a decrease in colonic hypersensitivity assessed 60 min after injection of 0.75% acetic acid in the urinary bladder and intrathecal administration of APETx2 was observed for distensions of 40 mmHg (*p* < 0.05) and 60 mmHg (*p* < 0.01; Figure 5) compared to control animals.

## 4. Discussion

In the present study, we successfully developed a non-inflammatory rat model of urinary bladder/colon cross-organ sensitization. Indeed, ultrasonography-guided administration of acetic acid into the urinary bladder of rat resulted in increased nociceptive responses to colorectal distension that lasted up to 3 days. Moreover, we showed in rats that ASIC-3 expressing primary extrinsic afferent neurons were more likely to co-innervate urinary bladder and colon, and subsequently showed that blocking ASIC-3 prevented acetic acid-induced cross-organ sensitization. To our knowledge, this is the first study showing that ASIC-3 channels may be involved in urinary bladder/colon cross-organ sensitization.

Traditionally, intravesical administration of intraluminal irritative agents is performed through laparotomy (6, 22, 23). In addition, measurement of colonic pain is based on the quantification of muscle contractions of the abdominal wall in response to CRD, a method also named pseudo-affective reflex (24), after surgical insertion of electromyography recording electrodes. However, and as we have previously shown, stress as well as surgery could by themselves promote visceral hypersensitivity (25). Here we aimed at developing an acute, non-inflammatory model of urinary bladder/colon cross-organ sensitization using mini-invasive techniques. In our model, intravesical administration of acetic acid was performed through percutaneous injection under brief anesthesia using ultrasound guidance. In addition, measurement of intracolonic pressure was performed as a reflection of intra-abdominal pressure, and therefore abdominal contractions to measure colonic viscero-sensitivity (25). This technique does not require surgery and has therefore the advantage to be minimally invasive and to prevent surgery-induced visceral hyperalgesia (25). By employing these approaches, we were able to record the transient colonic hypersensitivity (no longer than 3 days in duration) resulting from the instillation of acetic acid. Furthermore, we observed no significant difference in colonic permeability through Ussing chambers. This therefore translates that the colonic permeability was not impaired after an intravesical injection of acetic acid at 0.75%. Similarly, no significant difference of MPO activity in the colon and urinary bladder was observed in our model. This suggested that the observed cross-organ sensitization model was neither related to inflammatory processes nor to colonic hyperpermeability.

We also show in this study that an average of 9.7% of urinary bladder and colon primary afferent neurons in the L6 and S1 DRG co-innervate the urinary bladder and colon in Rat. These values agree with previous descriptions by Christianson and collaborators (2006) (26), also in Rat. In another study higher proportions of neurons co-innervating the urinary bladder and colon were shown (27). Indeed, these authors have shown that 14.0 ± 2.5% of neurons in L6 DRG and 14.3 ± 1.9% of neurons in the S1 DRG innervate both the colon and the urinary bladder in rats. However, the animals used in the later study were female rats (27). Whether there is gender difference in the proportion in neurons co-innervating the urinary bladder and the colon remains to be confirmed, although this may explain to some extent the higher prevalence of IBS and BPS in women.

To the best of our knowledge, this may be the first study addressing the neurochemical phenotype of dually projecting colorectum-urinary bladder DRG neurons. Here we show for the first time that neurons co-innervating the urinary bladder and the colon express ASIC-3 protein, and that the proportion of such neurons expressing this channel was larger than what could be observed in urinary bladder-only or colorectum-only DRG neurons. ASIC-3 has been shown to participate in mechanisms of visceral hypersensitivity. Thus, ASIC-3 appears to be involved in the induction of chronic colorectal hypersensitivity due to intracolonic zymosan in mouse, by sensitization of mechanoreceptors in the absence of inflammation (28). It has also been proposed that ASIC-3 contributes to the development of functional hypersensitivity, observed in patients with IBS (29). In another study, an increase in ASIC2a and ASIC3 mRNAs in the urinary bladder of BPS patients was observed, suggesting involvement of these channels in increased pain and hyperalgesia (30). In our present study, we also show that ASIC-3 seems to participate in mechanisms of urinary bladder-colorectal cross-organ sensitization. Rats given intrathecal APETx2, show that this ASIC-3 blocker could potentially prevent the occurrence of colonic hypersensitivity induced by vesical insult. However, there are some limitations of APETx2. In fact, we cannot exclude an effect of this blocker on the voltage-gated sodium (NaV) channel, NaV1.8 (31). On the other hand, multiple studies have showed that intrathecal injection diffuses in DRGs by using dye (32) or isotope (33). In addition, Marger et al. observed that intrathecal injection is much better than an intra-organ injection or an intraperitoneal injection (34). By injecting APETx2 intrathecally it was not intended to target central nervous system (CNS) cells, since ASIC-3 are not expressed in the CNS (35, 36). The aim was rather to target DRG cells as did other groups (37). Furthermore, it was showed in a genetic model of ASIC3 knockout in mice an increase in visceral nociceptive mechanical threshold (38, 39). These data suggest that ASIC3 may also play a role in colorectal mechanosensation under non-pathophysiological conditions. Interestingly, Jones et al. have also demonstrated that amiloride, an ASIC non-specific blocker applied directly on mucosal fields of a specific class of stretch-sensitive colonic afferents (muscular-mucosal afferents) in ex-vivo preparations obtained from control mice, did not affect mechanosensitivity (39). Nevertheless, these data were obtained from an ex-vivo model and amiloride do not have the same pharmacological action as APETx2 (40). In contrast, in a non-visceral study, it was demonstrated that APETx2 maintains the ASIC3 current amplitude unaltered (41).

In our study, we believe that APETx2 potentially acts as a preventive molecule of cross-organ sensitization through indirect inhibition of AIC3 upregulation in DRGs as it was observed in several studies (42, 43). Nevertheless, this statement remained to be demonstrated in our model.

In conclusion, we present here an alternative, minimally invasive model of urinary bladder insult that allows for instillation of different types of molecules without exposing the animal to excessive stress derived from surgical interventions. We also show that dually projecting colorectal and urinary bladder DRG neurons express ASIC-3, and this acid sensing channel may be relevant in the mechanisms of cross-organ sensitization between pelvic organs; this could represent a potential interesting therapeutic target for the future treatment of IBS/BPS syndromes which are characterized by the presence of chronic pain in most patients. However, as far as we know in the literature, there is no chronic non-inflammatory urinary bladder-colon cross sensitization model. Taken this fact into consideration, acute model is not a perfect model but remains the best one. Acute cross-organ sensitization in the context of IBS/BPS can also be seen as a possible trigger to enter both diseases (rather than a mechanism of sustained pain in the long run): indeed, 10% of IBS and/or BPS have inflammatory/chemical insult triggering their symptoms, including gastroenteritis, cystitis, IBD, and even stress that can impair the intestinal/urothelial permeability (44). Nevertheless, these results will require further confirmation and expansion, including if, for example, dually-projecting DRG neurons also express vanilloid receptors 1 (TRPV1). In an indirect fashion, it has been shown that the instillation of 4,6-trinitrobenzenesulphonic acid (TNBS) into the colon results in an increase of TRPV1 in S1 DRG neurons, and that urinary bladder nociceptive responses could be enhanced in part by TRPV1 activation in the urinary bladder (27). This and other molecules remain to be studied in non-inflammatory models of cross-organ sensitization.

## Data Availability

The original contributions presented in the study are included in the article/**Supplementary Material**, further inquiries can be directed to the corresponding author.
