# Supplementary figures and images for: Involvement of acid sensing ion channel (ASIC)-3 in an acute urinary bladder-colon cross sensitization model in rodent
Source: Front Pain Res (Lausanne). 2023 Mar 8;4:1083514. doi: 10.3389/fpain.2023.1083514 (PMC10030710; doi:10.3389/fpain.2023.1083514)

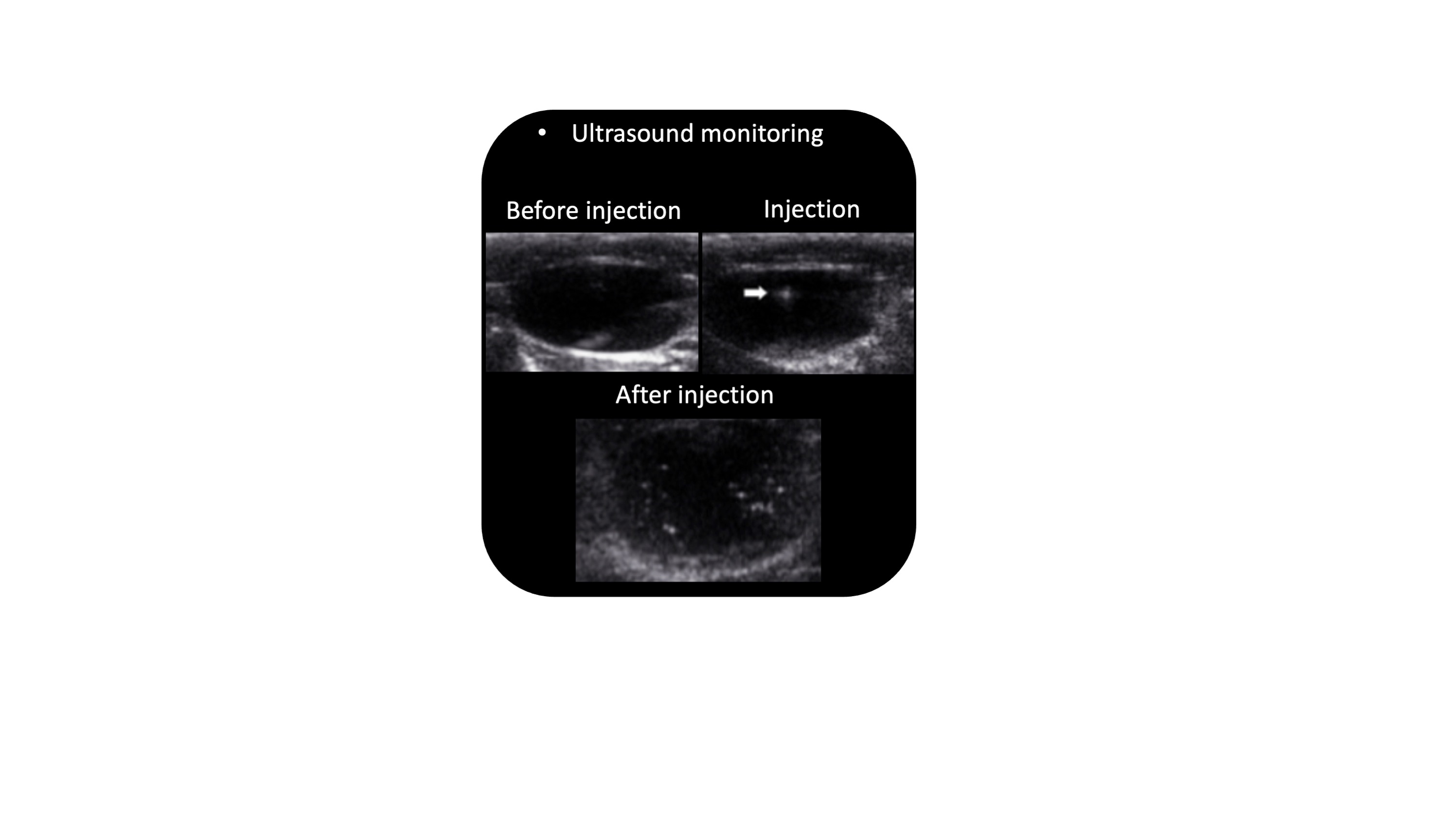

Supplement: Supplementary file 1 [file Image1.jpeg]
